# The association between CCND1 G870A polymorphism and colorectal cancer risk: A meta-analysis: Erattum

**DOI:** 10.1097/MD.0000000000009904

**Published:** 2018-02-09

In the article, “The association between CCND1 G870A polymorphism and colorectal cancer risk: A meta-analysis”,^[1]^ which appeared in Volume 96, Issue 42 of *Medicine*, the affiliation of Mei Xie should be “Chengdu University of Traditional Chinese Medicine.”
